# Potential Synergistic Effects of Caffeine and Naringin on Mitochondrial Biogenesis and Hepatic Steatosis in Adult Male Rats With NAFLD Induced by a High-Fat Diet

**DOI:** 10.1155/bmri/1565994

**Published:** 2025-08-13

**Authors:** Mehrasa Azizollahi, Zahra Nasehi, Maryam Derakhshan, Fouzieh Zadhoush

**Affiliations:** ^1^Department of Clinical Biochemistry, School of Pharmacy and Pharmaceutical Sciences, Isfahan University of Medical Sciences, Isfahan, Iran; ^2^Department of Pathology, School of Medicine, Isfahan University of Medical Sciences, Isfahan, Iran

**Keywords:** caffeine, high-fat diet, mitochondrial biogenesis, naringin, non-alcoholic fatty liver disease, non-esterified fatty acids

## Abstract

**Introduction:** Nonalcoholic fatty liver disease (NAFLD) is one of the most common causes of chronic liver disease worldwide, and disturbances in lipid metabolism and mitochondrial function play a significant role in its progression. In this study, to improve the effects of caffeine (CAF) treatment, we evaluated the effects of CAF and naringin (NAR) alone and in combination on gene expression involved in mitochondrial biogenesis, plasma nonesterified fatty acid (NEFA) levels, hepatic TG levels, and pathological changes in the liver tissue in adult male rats with NAFLD induced by a high-fat diet (HFD).

**Materials and Methods:** Then, 35 male Wistar rats were randomly assigned to five groups: control, HFD, HFD + CAF, HFD + NAR, and HFD + CAF + NAR (seven rats per group). They were fed a HFD containing 51% fat for 10 weeks, followed by a 6-week gavage treatment with CAF (50 mg/kg/day) and NAR (12.5 mg/kg/day), either individually or in combination. Gene expression related to mitochondrial biogenesis (SIRT1, PGC1*α*, and TFAM), serum NEFA levels, hepatic triglyceride (TG) levels, and liver histological changes were assessed.

**Findings:** The combination of CAF and NAR in the HFD + CAF + NAR group significantly increased the expression of SIRT1 (*p* < 0.01), PGC1-*α* (*p* < 0.01), and TFAM (*p* < 0.05) compared to the HFD group, while single treatments did not show such effects. Serum NEFA levels did not change significantly in any of the groups (HFD and treatment groups), but liver TG levels were significantly reduced in both single and combination treatments (*p* < 0.001). Pathological changes, including improvements in steatosis, inflammation, and ballooning, were observed in the treatment groups, particularly in the HFD + CAF + NAR group.

**Conclusion:** Based on current findings, the combined use of CAF and NAR as an adjunct therapy may exert its protective effects by enhancing the expression of genes involved in mitochondrial biogenesis, improving liver lipid levels, and ameliorating liver pathology. Therefore, it can be considered an innovative strategy for improving liver metabolic status in the context of NAFLD.

## 1. Introduction

Nonalcoholic fatty liver disease (NAFLD) is among the most prevalent liver diseases worldwide, affecting an estimated 32%–38% of adults, with men exhibiting higher rates than women. Without effective interventions, its prevalence is expected to rise significantly by 2030 [1, 2].

The underlying mechanisms of NAFLD involve hepatic lipid accumulation, mitochondrial dysfunction, and oxidative stress. The buildup of triglycerides (TGs) and free fatty acids (FFAs) in the liver leads to mitochondrial impairment and increased production of reactive oxygen species (ROS), further intensifying oxidative stress. Additionally, endoplasmic reticulum stress and lipotoxic compounds, such as saturated fatty acids and ceramides, play a crucial role in disease progression [3]. Mitochondrial dysfunction plays a crucial role in NAFLD progression, characterized by impaired fatty acid oxidation, reduced respiratory chain activity, excessive ROS generation, increased hepatic fatty acid influx, and enhanced fatty acid synthesis [4]. These metabolic alterations disrupt liver energy homeostasis and accelerate disease development [5].

Since mitochondrial dysfunction is central to NAFLD progression, improving mitochondrial quality through the upregulation of mitochondrial biogenesis offers a promising strategy for disease mitigation [6]. Through mitochondrial biogenesis, existing mitochondria grow and divide to ensure their maintenance and expansion [7]. The genes SIRT1, PGC-1*α*, and TFAM play crucial roles in regulating this process [7, 8]. SIRT1, an NAD+-dependent enzyme, deacetylates and activates PGC-1*α*, promoting the expression of mitochondrial genes and modulating energy metabolism. PGC-1*α*, in turn, upregulates TFAM, a critical factor for mitochondrial DNA transcription and organization [9].

The main pharmacological treatments for NAFLD include pioglitazone, vitamin E, and metformin, which contribute to reducing fibrosis, steatosis, and inflammation as well as enhancing insulin sensitivity. Furthermore, emerging drugs like sodium-glucose co-transporter 2 (SGLT2) inhibitors and obeticholic acid are currently under investigation. However, these emerging treatments may be associated with potential side effects, such as heart failure and an increased risk of mortality [10]. Given the lack of FDA-approved medications for NAFLD and the potential side effects of current treatments, alternative therapies are being investigated to enhance patient quality of life while maintaining efficacy and reducing adverse effects. Natural products provide notable benefits compared to synthetic chemical drugs, including fewer side effects, lower long-term toxicity, and greater diversity in bioavailability and biological activity [11]. Caffeine (1,3,7-trimethylxanthine) is one of the most widely consumed pharmacologically active compounds globally and is present in over 60 plant species, including cacao beans and tea leaves. It is also known as guaranine, theine, and mateine [12]. A 2017 meta-analysis assessing the relationship between coffee consumption and various health outcomes suggests that coffee may protect against liver disorders [13], lower the risk of developing NAFLD, and slow the progression of liver fibrosis in NAFLD patients [14].

Studies have shown that consuming caffeine at a concentration of 0.5 mg/mL in drinking water for 16 weeks provides significant protection against a high-fat diet (HFD) induced by NAFLD [15]. Moreover, Pu-erh tea extract (PTE) prevented NAFLD development in HFD-fed mice, while caffeine-free PTE failed to produce similar results [16]. While low doses of caffeine (under 400 mg daily) are widely regarded as safe for healthy adults, it is not entirely risk-free and, in excessive amounts, can cause serious toxicity or even death, often due to myocardial infarction or arrhythmia [17]. Additionally, caffeine is mainly metabolized in the liver by cytochrome P450 1A2 (CYP1A2) through an initial N3-demethylation, accounting for approximately 95% of its metabolism [18]. Consequently, natural CYP1A2 inhibitors that enhance caffeine's effects without requiring higher doses may hold potential for liver disease treatment.

Naringin (C₂₇H₃₂O₁₄) and its aglycone form, naringenin, are flavonoids commonly present in citrus fruits such as oranges, lemons, tangerines, and grapefruits. Upon ingestion, naringin is metabolized into naringenin in the intestine [19, 20]. These compounds function as competitive inhibitors of CYP1A2, extending caffeine's half-life and enhancing its effects [21]. Additionally, these compounds exhibit hepatoprotective and antifibrotic effects [19, 20].

Zhang et al. discovered that naringin enhanced lipid metabolism in tissue-engineered fatty (TEF) livers by decreasing fatty acid uptake and de novo lipogenesis, along with enhancing fatty acid oxidation [19]. Another study demonstrated that administering 3% naringenin (wt/wt) for 12 weeks significantly enhanced PGC1-*α* gene expression in mice consuming a high-fat, high-cholesterol diet for 12 weeks [22].

Due to the restricted efficacy of monotherapy in treating NAFLD, combination therapies have been developed to improve efficacy and reduce side effects by decreasing the dosage of the primary drug [23]. While caffeine and naringin have demonstrated antioxidant, anti-inflammatory, antiaging, and potential liver-protective properties, their combined impact on mitochondrial biogenesis and hepatic steatosis in NAFLD remains unexplored. This study is aimed at addressing this gap by investigating their influence in a HFD-induced rat model.

## 2. Materials and Methods

### 2.1. Experimental Animals

Then, 35 adult male Wistar rats, aged 8 weeks and weighing between 190 and 230 g, were obtained from the Animal Laboratory of the Faculty of Pharmacy and Pharmaceutical Sciences at Isfahan University of Medical Sciences, Isfahan, Iran. They were housed in standard polycarbonate cages (four rats per cage) under controlled conditions, including a temperature of 22 ± 1°C, 55% humidity, and a 12-h light/dark cycle, with free access to food and water. The study protocol and all animal procedures were approved by the Ethics Committee of Isfahan University of Medical Sciences.

### 2.2. Development and Classification of HFD-Induced NAFLD in Rats

The rats were divided into two dietary groups: one receiving a normal-fat diet and the other a commercially prepared HFD (containing 51% fat) ad libitum for 10 weeks. The HFD was obtained from the Razi Vaccine and Serum Research Institute (Tehran, Iran).

Following this period, the animals were divided into five groups (*N* = 7) as follows:

• Group I (control): Healthy rats received physiological saline orally for 6 weeks.
• Group II (HFD): HFD-fed rats received physiological saline by oral gavage for 6 weeks.
• Group III (HFD + CAF): HFD-fed rats received caffeine (50 mg/kg) dissolved in saline by oral gavage for 6 weeks [24].
• Group IV (HFD + NAR): HFD-fed rats received naringin (12.5 mg/kg) dissolved in saline by oral gavage for 6 weeks [25].
• Group IV (HFD + CAF + NAR): HFD-fed rats received both caffeine (50 mg/kg) and naringin (12.5 mg/kg) dissolved in saline by oral gavage for 6 weeks.

Following the treatment period, the rats were anesthetized with carbon dioxide, and blood samples were drawn through cardiac puncture. The samples were centrifuged at 3500 RPM for 10 min, after which the serum was separated and stored at −20°C in Eppendorf tubes for NEFA analysis. Liver tissues were isolated and split into two sections: one was snap-frozen in liquid nitrogen for TG content assessment and gene expression analysis of PGC1-*α*, SIRT1, and TFAM, while the other was preserved in 10% neutral formalin for histopathological evaluation.

### 2.3. Serum Nonesterified Fatty Acids (NEFAs)

Serum NEFA levels were assessed using the NEFA assay kit (Biorex Fars, Cat. No BXC0473), which employs the ACS-ACOD enzymatic colorimetric method. Absorbance was recorded at 546 nm at 37°C. The assay demonstrated linearity up to 4 mmol/L, with a minimum detectable concentration of 0.1 mmol/L.

### 2.4. Determination of Hepatic Lipid Content

Liver TG levels were determined using the Folch method [26]. A 50-mg tissue sample was homogenized with sodium sulfate, followed by the addition of methanol and chloroform. After overnight incubation at 4°C, phase separation was induced using a sodium chloride solution. The lipid-containing chloroform layer was extracted, evaporated with nitrogen gas, and dissolved in 2-propanol. The resulting solution was centrifuged, and the clear supernatant was collected for analysis. TG levels were quantified using the Triglyceride Assay Kit (Bayerx Fars, Cat. No. BXC0271), employing the GOD-PAP enzymatic method. Absorbance was recorded at 546 nm at 37°C, with a linear detection range up to 1000 mg/dL and a minimum sensitivity of 30 mg/dL.

### 2.5. Real-Time Quantitative PCR (qPCR) for Gene Expression Analysis

Total RNA was extracted from liver tissue using the RNX-Plus Solution (SinaClon, Tehran, Iran), following the manufacturer's instructions. The concentration and purity of RNA were evaluated using the 260/280 absorbance ratio on a Nanodrop Hybrid reader (Synergy H1, BioTek, United States), with a ratio between 1.8 and 2 indicating sufficient purity. Following RNA extraction, cDNA synthesis was carried out using the SinaClon First Strand cDNA Synthesis Kit (SinaClon, Tehran, Iran). The relative mRNA expression was quantified using the high ROX Amplicon Master Mix with SYBR Green (Amplicon, Denmark) on the StepOne Real-Time PCR System (Applied Biosystems, ABI, United States) and analyzed using the *ΔΔ*CT method, with GAPDH serving as the reference gene. The forward and reverse primer sequences (5⁣′ →3⁣′) are shown in Table 1.

The thermal cycling protocol began with an initial denaturation at 95°C for 15 min, followed by 40 amplification cycles. Each cycle included denaturation at 95°C for 15 s, annealing at 59°C for 30 s, and extension at 72°C for 30 s. To confirm the specificity of the amplified products, a melting curve analysis was performed by gradually increasing the temperature from 65°C to 95°C in 0.3°C increments.

### 2.6. Liver Histopathology Evaluation

Liver tissues were fixed in 10% formalin, embedded in paraffin, and sectioned into 5-*μ*m slices. The sections were stained with hematoxylin and eosin (H&E), and pathological changes were assessed by a histologist using a light microscope (Nikon, Japan) [27]. The histopathological scores for NAFLD are presented in Table 2 [28].

### 2.7. Statistical Analysis

Data are shown as means ± SD, except where noted as mean ± SEM. For comparisons between two groups, an independent-sample *t*-test was employed, while for comparisons involving more than two groups, a one-way analysis of variance (ANOVA) followed by Tukey's post hoc test was performed. Statistical analysis was conducted using SPSS 26.0 software, and a *p* value ≤ 0.05 was considered statistically significant.

## 3. Results

### 3.1. Body Weight Changes

**Figure 1:**
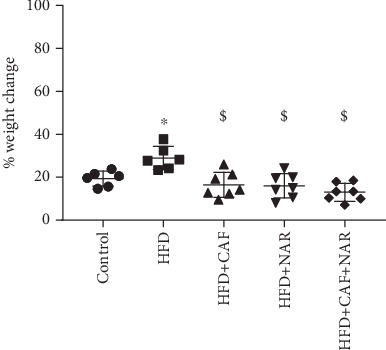
Comparison of body weight change among the healthy control, HFD, HFD + CAF, HFD + NAR, and HFD + CAF + NAR groups. HFD: high-fat diet group; HFD + CAF: high-fat diet group receiving caffeine (50 mg/kg/day); HFD + NAR: high-fat diet group receiving naringin (12.5 mg/kg/day); HFD + CAF + NAR: high-fat diet group receiving a combination of caffeine and naringin. Results are expressed as mean ± SD. ⁣^∗^*p* < 0.05 vs. the control and ^$^*p* < 0.05 vs. HFD.

As shown in Figure 1, feeding with a HFD significantly increased the percentage of body weight change in animals compared to the control group (*p* < 0.05). Then, 6 weeks of treatment with caffeine (50 mg/kg) (*p* < 0.05) and naringin (12.5 mg/kg) (*p* < 0.05), administered individually or in combination (HFD + CAF + NAR) (*p* < 0.05), significantly reduced the percentage of body weight change in rats compared to the HFD group.

### 3.2. Serum NEFAs

**Figure 2:**
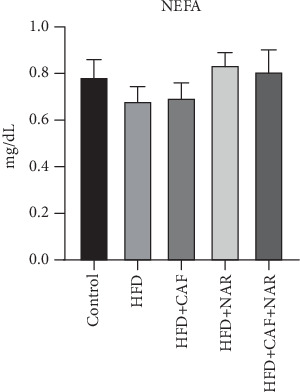
Comparison of serum nonesterified fatty acid (NEFA) levels in the healthy control, HFD, HFD + CAF, HFD + NAR, and HFD + CAF + NAR groups. HFD: high-fat diet group; HFD + CAF: high-fat diet group receiving caffeine (50 mg/kg/day); HFD + NAR: high-fat diet group receiving naringin (12.5 mg/kg/day); HFD + CAF + NAR: high-fat diet group receiving a combination of caffeine and naringin. Results are expressed as mean ± SD.

Measurement of serum NEFA levels showed no significant differences between the HFD and healthy control groups (*p* > 0.05), and no significant changes were noted in the treatment groups when compared to the HFD group (*p* > 0.05) (Figure 2 and Table 3).

### 3.3. Liver TG Levels

**Figure 3:**
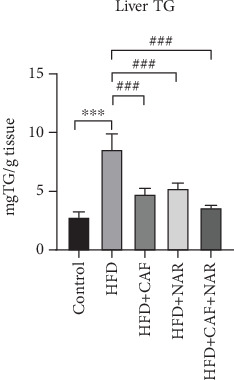
Comparison of liver tissue TG levels in the healthy control, HFD, HFD + CAF, HFD + NAR, and HFD + CAF + NAR groups. HFD: high-fat diet group; HFD + CAF: high-fat diet group receiving caffeine (50 mg/kg/day); HFD + NAR: high-fat diet group receiving naringin (12.5 mg/kg/day); HFD + CAF + NAR: high-fat diet group receiving a combination of caffeine and naringin. Results are expressed as mean ± SD. ⁣^∗∗∗^*p* < 0.001 vs. the control and ^###^*p* < 0.001 vs. HFD.

Results showed that a HFD for 10 weeks caused a marked increase in liver TG levels in the HFD group compared to the healthy control group (*p* < 0.001). Treatment with caffeine and naringin, either alone or in combination, significantly lowered liver TG levels compared to the HFD group (*p* < 0.001(. There was no significant difference in hepatic TG levels among the caffeine, naringin, and combined (CAF + NAR) treatment groups (Figure 3 and Table 3).

### 3.4. Expression of SIRT1, PGC1-*α*, and TFAM Genes

**Figure 4:**
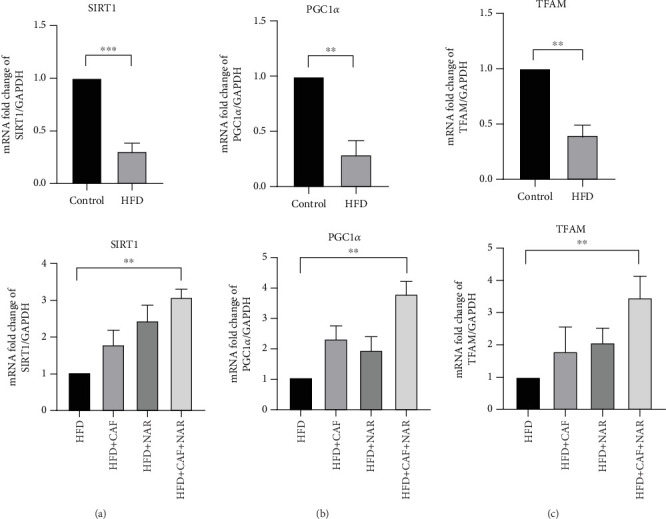
Relative gene expression levels of (a) SIRT1, (b) PGC1-*α*, and (c) TFAM in the healthy control, HFD, HFD + CAF, HFD + NAR, and HFD + CAF + NAR groups. HFD: high-fat diet group; HFD + CAF: high-fat diet group receiving caffeine (50 mg/kg/day), HFD + NAR: high-fat diet group receiving naringin (12.5 mg/kg/day); HFD + CAF + NAR: high-fat diet group receiving a combination of caffeine and naringin. Results are expressed as mean ± SD. ⁣^∗∗^*p* < 0.01 and ⁣^∗∗∗^*p* < 0.001.

According to Figure 4, the analysis of SIRT1, PGC1-*α*, and TFAM gene expressions revealed that a 10-week HFD led to a significant decrease in SIRT1 (*p* < 0.001, Figure 4a), PGC1-*α* (*p* < 0.01, Figure 4b), and TFAM (*p* < 0.01, Figure 4c) expressions compared to the healthy control group. Neither caffeine nor naringin treatment alone had a significant effect on SIRT1, PGC1-*α*, and TFAM gene expressions compared to the HFD group (*p* > 0.05). The combined treatment of caffeine and naringin resulted in a significant elevation of SIRT1 (*p* < 0.01, Figure 4a), PGC1-*α* (*p* < 0.01, Figure 4b), and TFAM (*p* < 0.01, Figure 4c) gene expressions compared to the HFD group.

### 3.5. Histopathology of Tissue Samples

**Figure 5:**
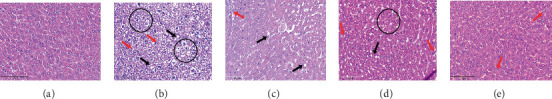
Pathological features of liver tissue in the healthy control, HFD, HFD + CAF, HFD + NAR, and HFD + CAF + NAR groups. Microscopic images of liver tissue from different groups include the following: (a) healthy control, (b) HFD, (c) HFD + CAF, (d) HFD + NAR, and (e) HFD + CAF + NAR. Magnification: 40×; scale bar = 100 *μ*m. Black arrows point to lipid droplets, red arrows highlight inflammation, and circles indicate ballooning damage.

**Figure 6:**
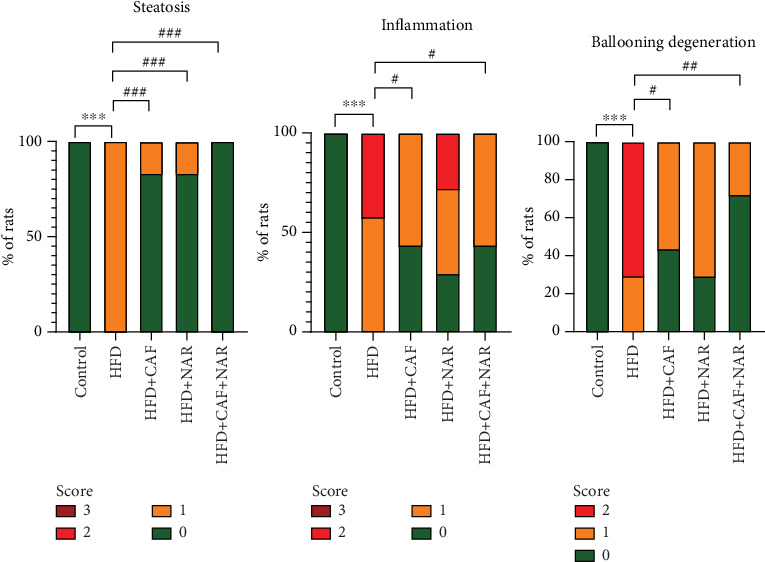
Comparison of steatosis, ballooning, and inflammation levels among the healthy control, HFD, HFD + CAF, HFD + NAR, and HFD + CAF + NAR groups. HFD: high-fat diet group; HFD + CAF: high-fat diet group receiving caffeine (50 mg/kg/day); HFD + NAR: high-fat diet group receiving naringin (12.5 mg/kg/day); HFD + CAF + NAR: high-fat diet group receiving a combination of caffeine and naringin. Results are expressed as mean ± SEM (⁣^∗∗∗^*p* < 0.001 vs. the control and ^###^*p* < 0.001 vs. HFD).

According to Figure 5, histopathological examination of liver tissues in the healthy control group (Figure 5a) revealed a normal structure, with zero scores for NAFLD-related changes. H&E staining showed Grade 1 steatosis, inflammation, and ballooning in the NAFLD group (Figure 5b). Following 6 weeks of treatment, caffeine (Figure 5c) and naringin (Figure 5d) individually reduced steatosis to a comparable degree, although their effects on inflammation and ballooning were different. The combined caffeine and naringin treatment (Figure 5e) resulted in significant improvements in all three areas: steatosis, ballooning, and inflammation. Statistical analysis results are shown in Figure 6.

## 4. Discussion

In this study, combined treatment with caffeine and naringin significantly improved histopathological findings and the expression of mitochondrial biogenesis-related genes. Although hepatic TG levels did not differ significantly compared to single treatments, other results indicated greater efficacy of the combination therapy.

Our findings revealed that after 10 weeks on a HFD, the hepatic TG levels in the HFD group were three times higher than those in the normal group, while no significant change in serum NEFA levels was observed. Furthermore, although no difference was found in serum NEFA among the treatment groups, liver TG levels were significantly reduced in the combined treatment group compared to the HFD group. These findings contrast with some previous studies.

Wang et al. reported that a 12-week HFD led to a significant increase in serum NEFA levels, as well as elevated serum and hepatic TG levels, in the HFD group compared to the control group [29]. Similarly, another study demonstrated that a 12-week HFD led to elevated NEFA levels in the HFD group compared to the control group [30]. On the other hand, consistent with our findings, a study on LDL receptor-deficient mice found no significant alteration in plasma NEFA levels following 1% and 3% naringin treatment under both normal and HFD conditions [31]. Likewise, another study reported no significant variation in serum NEFA levels between the HFD and control groups after a 3-week HFD, although liver TG levels were elevated in the HFD group [32]. Gauthier et al. stated that 16 weeks of a HFD did not result in a significant change in plasma NEFA levels, which they attributed to the body's metabolic adaptations, such as increased fat oxidation capacity and a possible enhancement in adipose tissue lipid storage [33].

We agree with the hypothesis that the liver sequesters and stores circulate NEFA as TG, which could account for the absence of significant differences in NEFA levels between the study groups [34, 35]. TG accumulation might act as a defense mechanism in the transition from NAFLD to NASH by storing NEFA as TG, thereby shielding the liver from lipotoxic damage. However, as the disease advances, this protective function diminishes, and the liver's impaired ability to synthesize TG leads to a significant rise in both serum and hepatic NEFA levels [34]. Thus, it is proposed that the liver damage caused by a HFD in this study may not have been extensive enough to produce a significant alteration in serum NEFA levels, requiring a longer duration of exposure [35].

Furthermore, our study revealed a significant reduction in SIRT1 expression in the HFD group. While treatment with either caffeine or naringin alone did not produce a notable effect, their combination significantly upregulated SIRT1 expression. As an NAD+-dependent deacetylase, SIRT1 plays a key role in regulating cellular processes and lipid metabolism, and its activation may contribute to reducing NAFLD severity [36].

In line with our findings, other studies have shown that a HFD decreases SIRT1 expression in the liver and reduces SIRT1 protein levels in the HFD group [37, 38]. In contrast to our findings, a 2024 study found that caffeine enhances the expression of SIRT1 and PGC-1*α* in kidney cells, thereby promoting mitochondrial biogenesis [39]. The differences in results may be due to the direct caffeine application in the 2024 study, which could lead to increased local concentrations, or the impact of the in vitro conditions on cellular behavior. Furthermore, Salama et al. demonstrated that naringin (300 mg/kg) offers protective effects on lung health and mitochondrial function through SIRT1 pathway activation [40]. The lack of protective effects and favorable gene expression changes is probably due to the low dose of naringin administered in this study, which may not have been strong enough to induce such effects. While no significant rise in SIRT1 gene expression was found in the caffeine or naringin-only groups, their combined treatment significantly boosted expression compared to the HFD group, likely reflecting a synergistic effect.

Our findings also indicate that the expression of the PGC1-*α* gene, a crucial regulator of mitochondrial biogenesis, was lower in the HFD group compared to the healthy control group. However, no significant difference in PGC1-*α* expression was observed in the HFD groups that received either caffeine or naringin alone. Previous studies have also reported decreased PGC-1*α* expression in fatty liver models [41, 42]. In a 2019 study, caffeine prevented the reduction of PGC1-*α* expression in rats on a HFD, although it did not raise expression to the same extent as in the group on a standard diet [43]. After 12 weeks of naringenin treatment, PGC1-*α* expression was increased in mice on a HFD, and this effect was similarly observed in both wild-type and Fgf21−/− mice after 16 weeks [22, 44]. Our study found that while caffeine and naringin alone did not notably enhance PGC1-*α* expression, their combination produced a significant increase, suggesting a synergistic effect.

Furthermore, our study showed that TFAM gene expression was significantly reduced in the HFD group compared to the healthy control group. While no significant increase in TFAM expression was seen in the groups treated with caffeine or naringin alone, the combined treatment led to a significant increase in TFAM expression compared to the HFD group.

TFAM is an essential protein involved in the transcription and structural organization of mtDNA, and it is widely recognized as a key marker of mitochondrial biogenesis [9]. In agreement with our findings, mice on a 12-week and 16-week HFD showed a significant decrease in TFAM expression [29, 45]. Unlike our findings, Schnuck et al. reported that a 12-h exposure to 50 *μ*M caffeine raised TFAM expression in muscle cells [46]. A study on NAFLD revealed that naringenin enhanced TFAM expression by upregulating PGC1-*α*, thereby promoting mitochondrial biogenesis in fat and muscle cells [25]. Another study demonstrated that a dose of 100 mg/kg of naringin significantly increased PGC1-*α* and TFAM expression in the hippocampus of male Wistar rats suffering from chronic tinnitus [47]. The absence [48] of a significant increase in TFAM gene expression in this study may be attributed to the short duration and low dosage of caffeine and naringin treatments when used individually. The combination of caffeine and naringin, however, significantly enhanced TFAM expression in the treatment group relative to the HFD group, pointing to a potential synergy between these two compounds. Based on the results, it can be concluded that a HFD reduces the expression of SIRT1, followed by PGC1-*α* and TFAM, while the combination of caffeine and naringin enhances the expression of these genes. The NAD+-dependent SIRT1–PGC-1*α*–TFAM pathway plays a crucial role in regulating mitochondrial function. Both SIRT1 and PGC-1*α* are located within the mitochondria, near mtDNA, where they regulate oxidative phosphorylation and activate TFAM transcription to protect mtDNA from oxidative damage caused by ROS [8].

Histological analysis further confirmed that the combined treatment of caffeine and naringin significantly alleviated hepatic steatosis, ballooning degeneration, and inflammation in the HFD groups. This was evident by a marked reduction in lipid droplets, swollen hepatocytes, and inflammatory cell infiltration. Although both compounds had beneficial effects on HFD when used separately, the combined treatment proved to be significantly more effective.

## 5. Conclusion

This study provides valuable insights into the effects of caffeine, naringin, and their combination in mitigating the adverse effects of a HFD, particularly with regard to liver health and mitochondrial function. The observed improvements in liver TG levels, gene expression related to mitochondrial biogenesis, and the expression of critical regulators such as SIRT1, TFAM, and PGC1-*α* highlight the potential of these compounds in combating NAFLD and promoting mitochondrial health. The combination of caffeine and naringin demonstrated synergistic effects, suggesting their complementary roles in improving cellular processes. Although further research, including longer treatment periods and different dosages, is warranted, the promising results from this study provide a strong foundation for future investigations into the therapeutic potential of caffeine and naringin in liver diseases and mitochondrial dysfunction. A graphical abstract summarizing the study findings is included in the Supplementary Information (available here).

## Figures and Tables

**Table 1:** Primer sequences used for real-time quantitative PCR (qPCR) analysis.

	**Forward**	**Reverse**
GAPDH	GGAAGCTGGTCATCAACGG	TCCACGACATACTCAGCACC
SIRT1	GGAACCTCTGCCTCATCTACA	GCATACTCGCCACCTAACCT
PGC1*α*	GTGGATGAAGACGGATTGCC	GTCAGGCATGGAGGAAGGAC
TFAM	TTGTCATTGGGATTGGGCAC	GCATTCAGTGGGCAGAAGTC

**Table 2:** The NASH CRN NAFLD activity score was used to assess the condition. Steatosis was evaluated based on the percentage of fat present in hepatocytes at 4× and 10× magnification.

**Steatosis (S) (%)**	**Lobular inflammation (L)**	**Hepatocyte ballooning (B)**
0: < 5%	**0**: none	**0**: none
1: 5%–33%	**1**: in < 2 foci	**1**: a few ballooned cells
2: 34%–66%	**2**: 2–4 foci	**2**: many ballooned cells
3: > 66%	**3**: > 4 foci	

**Table 3:** Serum nonesterified fatty acid (NEFA) and hepatic triglyceride (TG) levels in the healthy control, HFD, HFD + CAF, HFD + NAR, and HFD + CAF + NAR groups. HFD: high-fat diet group; HFD + CAF: high-fat diet group receiving caffeine (50 mg/kg/day); HFD + NAR: high-fat diet group receiving naringin (12.5 mg/kg/day); HFD + CAF + NAR: high-fat diet group receiving a combination of caffeine and naringin.

	**Control (** **n** = 7**)**	**HFD (** **n** = 7**)**	**HFD + CAF (** **n** = 7**)**	**HFD + NAR (** **n** = 7**)**	**HFD + CAF + NAR (** **n** = 7**)**
Serum NEFA (mg/dL)	0.76 ± 0.21	0.65 ± 0.18	0.68 ± 0.16	0.83 ± 0.15	0.81 ± 0.20
Liver TG (mg/g tissue) levels	2.77 ± 0.44	8.7 ± 1.4⁣^∗∗∗^	4.75 ± 0.62^###^	5.25 ± 0.58^###^	3.59 ± 0.26^###^

*Note:* Results are expressed as mean ± SD.

⁣^∗∗∗^*p* < 0.001 vs. the control group and ^###^*p* < 0.001 vs. the HFD group.

## Data Availability

Data are available on request.
