# Supplementary material for: Potential Synergistic Effects of Caffeine and Naringin on Mitochondrial Biogenesis and Hepatic Steatosis in Adult Male Rats With NAFLD Induced by a High-Fat Diet
Source: Biomed Res Int. 2025 Aug 13;2025:1565994. doi: 10.1155/bmri/1565994 (PMC12367387; doi:10.1155/bmri/1565994)
Supplement: Supporting Information — Additional supporting information can be found online in the Supporting Information section. The graphical abstract is provided as a supporting file. [file 1565994.f1.docx]

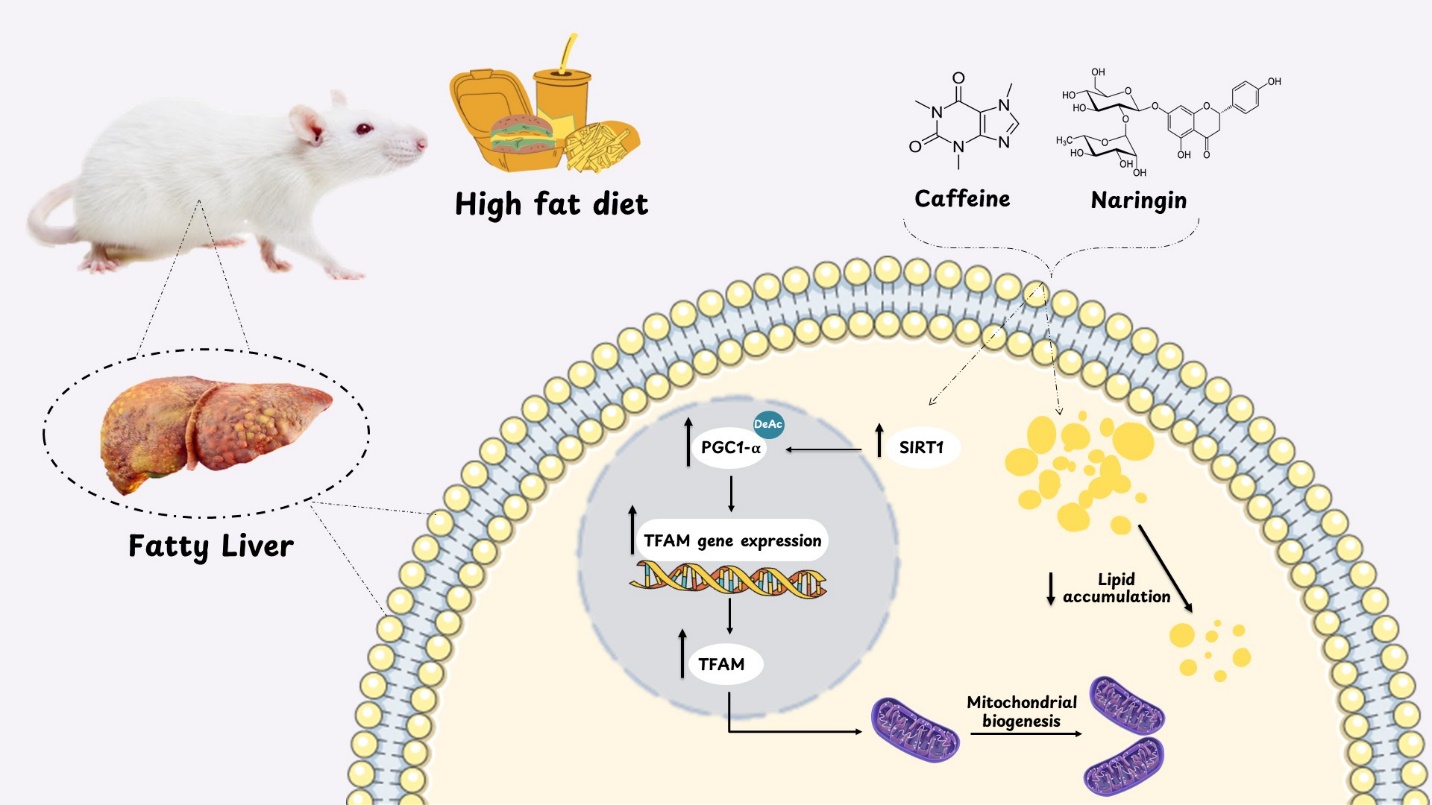


**Graphical Abstract**: Schematic representation of the combined effects of caffeine and naringin on mitochondrial biogenesis. The diagram illustrates the upregulation of SIRT1, PGC-1α, and TFAM, highlighting their roles in mitochondrial function and lipid metabolism
